# Emerging trends in cognitive impairment and dementia among older populations in Asia: A systematic review

**DOI:** 10.7189/jogh.14.04233

**Published:** 2024-11-08

**Authors:** Binish Islam, Tianjiao Li, Mengying Xu, Dan Yang, Hanxiao Lv, Goudja Gassara, Tasiu I Ibrahim, Bakeel A Radman, Jianwu Wang

**Affiliations:** 1 Xiangya School of Public Health, Central South University, Changsha, China; 2 Department of Neurological Surgery, Second Xiangya Hospital of Central South University, Changsha, China; 3 Department of Biology, College of Science and Education, Albaydha University, Albaydha, Yemen

## Abstract

**Background:**

Dementia and cognitive impairment rates in Asia have significant policy implications. Contrary to the existing literature, which primarily focused on the Western region, in this study, we provide novel insights into previously unexplored geographical contexts. We aimed to evaluate the prevalence of cognitive impairment and dementia in Asia.

**Methods:**

Adhering to the Preferred Reporting Items for Systematic Reviews and Meta-Analyses (PRISMA) 2020 guidelines, we searched six bibliographic databases: Web of Science, Medline, Science Direct, Ovid, Google Scholar, and PubMed. We targeted cross-sectional studies on dementia and cognitive impairment in Asia, published between 2019–23.

**Results:**

Our extensive search yielded 2593 original articles, of which 39 met eligibility criteria. This selection unveiled a significant rise in dementia and cognitive impairment prevalence in Asia, aligning now with trends observed in Western countries – a novel finding that challenges previous assumptions about regional prevalence disparities. The studies predominantly conducted in East Asia (n = 29), along with limited research from Southeast (n = 2), South (n = 7), and Central Asia (n = 1), underscore the geographical gaps in current research. This shift in prevalence patterns is potentially linked to demographic changes, urbanisation, environmental factors, ethnic diversity, and neuroimaging advancements. Identifying modifiable risk factors associated with dementia in these regions presents new avenues for prevention and intervention strategies.

**Conclusions:**

Current dementia research in Asia is concentrated in East Asia, with limited data from Southeast, South, and Central Asia. Comprehensive studies across all parts of Asia are crucial to establishing robust data collection methods and identifying modifiable risk factors. This can help manage and mitigate the growing burden of dementia in these societies.

The global demographic shift towards an ageing population presents a significant public health challenge, particularly the increasing prevalence of neurodegenerative diseases and functional limitations in older adults [1]. Among these, dementia stands out as a significant contributor to disability in the elderly population [2]. This multifaceted syndrome is characterised by a persistent and irreversible decline in cognitive function, significantly impairing the activities of daily living of older individuals [3]. Dementia is a progressive neurodegenerative disorder caused by a complex interaction of genetic, environmental, and behavioural variables [4]. It has a tremendous influence on social and physical activities, as well as general quality of life [5]. Cognitive abnormalities in disorders such as cognitive impairment and mild cognitive impairment are less severe than those in dementia [6]. Individuals with these conditions generally maintain their daily functions and independence [7]. However, these conditions are progressive, and in about one-third of cases, they are considered precursors to Alzheimer disease [8].

The incidence of dementia and cognitive disorders is globally trending upward [9]. This increase is predicted to be disproportionately higher in less developed countries than in developed ones [10–13]. The median age of the global population is expected to rise from 26.6 years in 2000 to 37.3 years in 2050 and 45.6 years by 2100 [14]. This rise in older population is estimated to occur predominantly in low- and middle-income countries [15]. Currently, approximately 57.4 million individuals are estimated to be living with dementia globally, a number expected to nearly double every two decades [16]. By 2050, around 152.8 million cases are projected, with a substantial proportion occurring in developing nations [13]. In 2010, 35.6 million people lived with dementia worldwide, and this number is expected to double every 20 years [17]. Notably, in 2010, 58% of all people with dementia were in low- or middle-income countries, a proportion projected to rise to 71% by 2050 [1]. Therefore, the anticipated increase in the prevalence of dementia in developing regions is expected to escalate both the health and social burdens in these areas [18].

The demographic and epidemiological transitions in developing countries, particularly in Asia, are leading to significant changes in health and disease patterns [19]. With increased population ageing, there is a rise in the burden of noncommunicable and degenerative diseases, including dementia and other cognitive disorders [20]. Earlier studies in Asian populations have reported a lower prevalence of dementia and cognitive impairment compared to studies conducted in Western populations [21]. However, recent research indicates a shift in this trend, reporting similar prevalence rates [22].

Despite socioeconomic, cultural, and health care variations across Asian countries, a comprehensive comparison remains relevant and valid as it provides a macroscopic view and identifies common risk factors [23]. There is a significant knowledge gap in Asia regarding the prevalence and impact of cognitive impairment and dementia, primarily due to outdated estimates and studies that often narrowly focus on individual countries or specific sub-regions [24]. Additionally, the diverse methodologies and diagnostic criteria used across these studies have resulted in inconsistent findings regarding prevalence rates and risk factors [25].

Considering these issues, we aimed to provide an updated analysis by incorporating studies published within a recent period (2019–23). In this systematic review, we focused on the latest trends and shifts in prevalence, which is crucial for accurately assessing current and future public health needs. We employed a consistent set of inclusion criteria and rigorous methodological standards to integrate findings across all sub-regions of Asia.

By undertaking this approach, we addressed the geographical gaps in existing research, providing a robust comparison of the prevalence of cognitive impairment and dementia across the continent. This comprehensive perspective is anticipated to yield novel insights into regional disparities in prevalence and associated risk factors, thereby significantly contributing to the global understanding of these conditions. These insights are crucial because of the rapidly ageing populations across Asia, which necessitates the need for tailored public health strategies and interventions.

## METHODS

### Search strategy

We conducted a systematic review according to the Preferred Reporting Items for Systematic Reviews and Meta-Analyses (PRISMA) guidelines [26], focusing on the prevalence of cognitive impairment and dementia in Asian countries. Our study approach involved a comprehensive search using specific keywords and subheadings to accurately analyse articles from six major bibliographic databases – Web of Science, Medline, Science Direct, Ovid, Google Scholar, and PubMed.

The scope of the review was broad, encompassing studies conducted across all 48 countries of Asia. To ensure the relevance and recency of the data, we limited our search to articles published within the past five years, between 2019–23. To augment our database search, we manually examined the retrieved articles’ reference lists to identify additional pertinent studies.

We used specific Medical Subject Headings terms for a more targeted search. These included combinations of ‘prevalence OR epidemiology’ and ‘dementia OR cognitive impairment’, paired with the names of each Asian country. We designed this approach to yield a comprehensive collection of studies relevant to our research question, covering a wide geographical and temporal span.

### Inclusion and exclusion criteria

We established specific inclusion and exclusion criteria to ensure the relevance and quality of the selected studies. The inclusion criteria were: original research articles related to the issue of this study; studies performed within the geographical borders of Asia; cross-sectional studies (we omitted books, qualitative studies, policy briefs, case studies, and other comparable publications); English-language studies; and peer-reviewed journal articles. Commentaries, reviews, and non-peer-reviewed research publications were eliminated.

The choice to include only cross-sectional studies was made to ensure methodological homogeneity and comparability of results across studies. Cross-sectional studies provide a consistent snapshot of prevalence at a specific time, allowing for direct comparisons and minimising variability introduced by different study designs.

### Data extraction

The data extraction process was thorough and systematic. Using the EndNote (Clarivate, London, UK) we first eliminated all duplicate articles. A preliminary screening based on the title and abstract of each article allowed for the initial removal of some selected articles. This was followed by a comprehensive reading of the complete texts, leading to selecting studies that strictly adhered to the inclusion/exclusion criteria.

Two independent reviewers reviewed the retained articles. Subsequently, any article meeting any of the exclusion criteria was rejected. From the selected articles, we extracted vital information, including the authors, country/region of the study, date of publication, screening tool and diagnostic criteria used, population age, study setting, sample size, and prevalence data.

### Quality assessment

To ascertain the methodological quality of the articles included in our review, we used a checklist created by the Joanna Briggs Institute, mainly for cross-sectional analytical studies [27]. The assessment of the quality of the included studies is provided in Table S1 in the **Online Supplementary Document**. This approach ensured a comprehensive and systematic evaluation of each article. Our analysis revealed uniformity across the studies in both approach and outcomes. Furthermore, according to the standards established by the JBI Meta-Analysis of Statistics Assessment and Review Instrument, all articles we reviewed were classified as high quality.

## RESULTS

### Study selection

**Figure 1:**
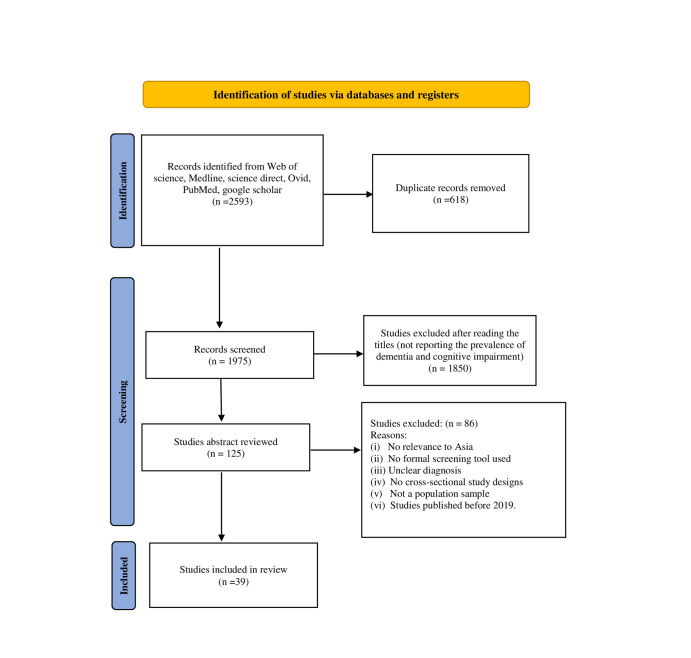
PRISMA flow diagram for the selection of studies.

Our systematic search across six databases initially identified 2593 original articles. We then applied several steps to refine this pool of articles to those most relevant to our research question. The first step of refining was the removal of duplicates, which reduced the number to 1975 articles. We then proceeded with a title-based screening, which led to a substantial reduction as 1850 articles were excluded, primarily due to their irrelevance to the Asian region. The next phase involved a detailed review of the abstracts of the remaining 125 articles. This stage was critical in assessing the suitability of the studies against our inclusion criteria, resulting in the exclusion of 86 more articles. The final stage entailed a comprehensive full-text review of the remaining articles. After this comprehensive evaluation, we identified 39 articles that met all the inclusion criteria and were deemed relevant for our systematic review (**Figure 1**). This thorough selection process reflects our review’s adherence to methodological standards.

**Figure 2:**
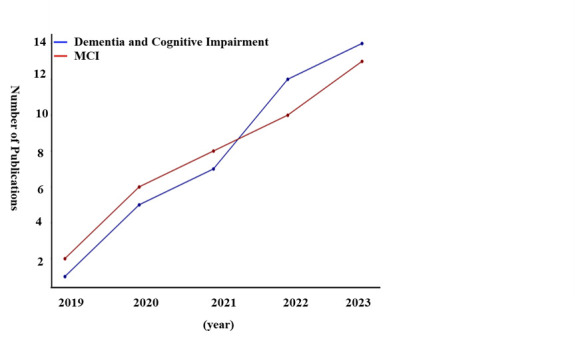
Temporal trend of publications (2019–23).

Following the systematic search and selection process outlined in the PRISMA flow diagram, further distribution of the identified studies over time to understand trends in research focus from 2019 to 2023. The temporal distribution of studies provides insights into the growing interest and research efforts in the fields of dementia, mild cognitive impairment (MCI), and cognitive impairment within Asia. It also reflects the evolving landscape of scientific inquiry as it adapts to increasingly recognising these conditions as major public health concerns. The line graph illustrates the annual count of publications that met our inclusion criteria, highlighting the year-on-year changes in research output across the study period (**Figure 2**).

### Overview of included studies

**Figure 3:**
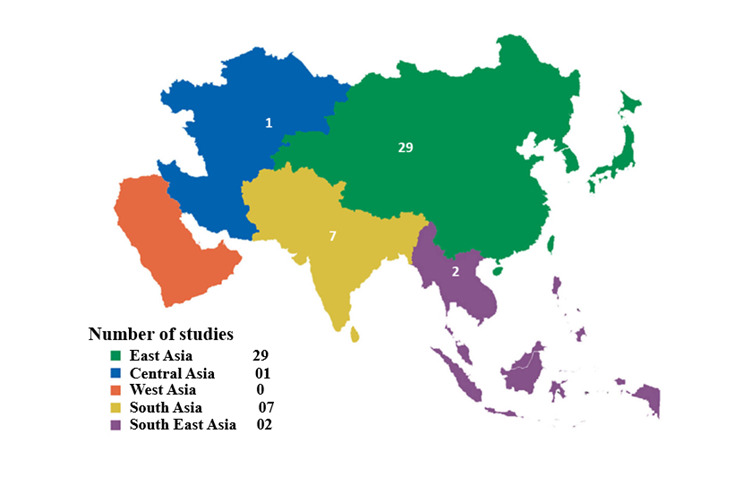
Map of distribution of cross-sectional studies in different sub-Asian regions.

The studies included in this systematic review have been summarised and grouped by sub-regions (**Table 1**). 29 studies were conducted in East Asia, two in Southeast Asia, seven in South Asia, and one in Central Asia (**Figure 3**). The sample size varied from 150 to 46 011 people and older adults (aged 50 to ≥65 years), except for one research, which included participants aged 20–97 years [33]. A total of five studies reported dementia with MCI, 10 studies reported cognitive impairment, 13 studies reported dementia, 10 studies reported MCI only, and one study reported dementia with cognitive impairment and MCI. 13 studies were conducted in urban settings, 13 studies were conducted in rural settings, and 13 were carried out in both rural and urban settings. These studies were all cross-sectional and published between 2019–23. Most studies applied a modified version of the Mini-Mental Status Examination as a screening tool. The Diagnostic and Statistical Manual of Mental Disorders and the International Classification of Diseases were the most widely used diagnostic tools for dementia. To provide a regional perspective, we categorised studies by their country of origin into Asian sub-regions – East Asia, South Asia, and Southeast Asia.

**Table 1:** Overview of included study characteristics

Author, region/country	Sample size (n)	Key inclusion criteria in years	Study purpose	Setting	Study measures	Diagnostic criteria	Prevalence of MCI, CI, and dementia
East Asia							
*Lor et al. [* 28 *], Taiwan*	4578	65	To explore how body measurements, physiological factors, chronic diseases, and social and lifestyle elements affect cognitive abilities in older adults living in Taiwanese communities.	Urban and rural	SPMSQ	SPMSQ≥3	CI 2.3%
*Kao et al. [* 29 *], Taiwan*	6549	76.98 (SD = 13.39)	To understand the current state of dementia.	Urban and rural	CDR, MMSE	CDR≥0.5, MMSE≤25	Dementia 87.1%
*Liu et al. [* 30 *], Taiwan*	10 432	≥65	To assess the differences in occurrence and risk factors for MCI and dementia between urban and rural areas.	Urban and rural	CDR	National Institute on Aging and Alzheimer Association	Dementia: rural (8.69%), followed by suburban (6.63%), and urban areas (4.46%). MCI for rural, suburban, and urban areas was 20.29%, 16.67%, and 15.11% respectively.
*Hsieh et al. [* 31 *], Taiwan*	4722	460 participants (≤65), 2113 participants (>65)	To study behavioural and psychological symptoms of dementia in institutionalised residents in Taiwan.	Urban and rural	MMSE, CDR	Very early dementia (CDR = 0.5), mild dementia (CDR = 1), severe dementia (CDR = 3), moderate dementia (CDR = 2)	MCI 18.76%, dementia 87.2%
*Ho et al. [* 32 *], Hong Kong*	2077	≥65	This research examined the link between public housing neighbourhood traits and dementia in Hong Kong’s elderly population.	Uraban and suburbs	Cantonese version of MoCA-B	Cognitive assessment ≤19	Dementia 41.4%
*Takenoshita et al. [* 33 *], Japan*	1831	20–97 (x̄ = 54.7)	To identify the frequency of dementia in individuals with intellectual disabilities excluding Down syndrome and to pinpoint the risk factors associated with dementia.	Urban and suburban	Japanese version of the Dementia Screening Questionnaire	ICD-10	Dementia 6.44%
*Shimizu et al. [* 34 *], Japan*	1220 (1997), 1290 (2004), 1129 (2012)	≥65	To study the changes over 20 y in the prevalence of all types of dementia and their specific forms in Japan.	Rural	MMSE	DSM-III-R, NINCDS-ADRDA	Increasing trend of dementia from 4.5% in 1997 to 9.5% in 2016
*Wang et al. [* 35 *], China*	1437	≥65	This research focused on the differences in CI between genders among seniors aged ≥65 y in rural China.	Rural	CMMSE	MMSE≤17 for illiteracy, MMSE = 20 for primary school, MMSE = 24 for secondary school	CI 40.0% in males and 45.1% in females
*Jiang et al. [* 36 *], China*	892	>50	To assess how supplementing with folic acid, B vitamins, vitamin D, and coenzyme Q10 affects cognitive ability.	Urban	MMSE, MoCA-B, ACE-III	NIA-AA clinical standard	CI 53.8%
*Cong et al. [* 37 *], China*	5068	≥60	The research centred on the epidemiological analysis of MCI and its variants in rural Chinese populations.	Rural	CDR, CMMSE	Neuropsychological assessments	CI 26.47%
*Gan et al. [* 38 *], China*	7528	x̄ = 74.85	To explore the link between sleep patterns and the occurrence of dementia with Lewy bodies in older Chinese adults.	Rural	Neuropsychological assessments	DSM-IV	Dementia 12.2%,
*Jiang et al. [* 39 *], China*	522	>60	To examine how common CI is and its connection with social support in the urban elderly population of Jinan, China.	Urban	MMSE	≤17 for illiterate participants, ≤20 for participants with primary education	CI 24.4%
*Liu et al. [* 40 *], China*	1325	≥60	To investigate the differences in MCI prevalence between genders and the relationship with various influencing factors.	Suburban	MMSE	≤17 for illiterate people, ≤20 for people with primary school, ≤24 for people with middle school or higher	MCI 15.2%, with 10.2% in men and 18.9% in women
*Liu et al. [* 41 *], China*	644 (Qinghai 207, Guangzhou 437)	x̄ = 78.1 (Qinghai), x̄ = 81.5 (Guangzhou)	To study how environmental aspects, particularly altitude, are related to CI in the elderly.	Urban and rural	MoCA-B	Score <26	CI 90.9%
*Xu et al. [* 42 *], China*	1262	≥65	To assess the link between dietary habits and the risk of MCI in rural elderly populations in China.	Rural	MMSE	MMSE for elderly people (16/17), MMSE for no education (19/20 for 1–6 y of education), MMSE 23/24 for >6 y of education	MCI 25%
*Xue et al. [* 43 *], China*	2146	≥60	To examine the frequency of MCI in China’s rural elderly and identify related risk factors.	Rural area	MoCA-B	Petersen criteria	MCI 23.16%
*Liu et al. [* 44 *], China*	2644	≥65	To contrast the occurrence of MCI and its various forms between urban and rural regions in Hubei Province, China.	Urban and rural	CDR, MMSE, MoCA-B	Petersen’s criteria	MCI 27.8%
*Ma et al. [* 45 *], China*	9036	≥65	To explore the occurrence of MCI in elderly people in rural northern China and its connection with hypertension and adherence to medication.	Rural	MMSE	MMSE<17 for illiterate subjects, MMSE<20 for subjects with 1–6 y of education, MMSE<24 for subjects with ≥7 y of education	MCI 18.1%
*Xu et al. [* 46 *], China*	2598	≥60	To evaluate the occurrence of CI in older adults in a specific region of China and investigate the related risk factors.	Urban and rural	CDR, MMSE	MMSE≤24, CDR≥0.5	CI 21.48%, MCI 15.70%, dementia 5.77%
*Fu et al. [* 47 *], China*	4943	≥60	To evaluate the frequency and risk factors of MCI among older adults in Tianjin, China, focusing on differences by age and gender.	Urban	MMSE	Petersen’s criteria	MCI 10.7%
*Fan et al. [* 48 *], China*	675	x̄ = 48.5	To explore the relationship between levels of blood non-esterified fatty acids, saturated fatty acids, and polyunsaturated fatty acids and MCI in the Chinese population aged 35–64 y.	Urban	MMSE, MoCA-B	MMSE = 19 (MCI illiterate individuals), MMSE = 26 (MCI educated individuals)	MCI 12.4%
*Duan et al. [* 49 *], China*	3111	≥60	To investigate the link between dietary patterns and MCI in elderly adults in China.	Rural area	MMSE	Modified Petersen’s criteria	MCI 11.70%
*Mima et al. [* 50 *], China*	9116	>50	To study the prevalence and risk factors associated with dementia.	Urban and rural	CMMSE	CMMSE<19 for illiterate, CMMSE≤19–22 for primary school, CMMSE≤22–26 for middle school and above	Dementia in males 5.12%, females 4.28%
*Song et al. [* 51 *], China*	6430	≥65	To find out how common dementia is in Xiamen, China, and to pinpoint the independent risk factors linked to it.	Urban and rural	MMSE	Score <27	Dementia 7.62%
*Jia et al. [* 52 *], China*	46 011	≥60	To assess the prevalence, risk factors, and management strategies for dementia.	Urban and rural	MMSE, MoCA-B, Hachinski Ischemic Score, CDR	DSM-IV	Dementia 6.0%, Alzheimer disease 3.9% MCI 15.5%, other dementias 0 · 5%
*Wang et al. [* 53 *], China*	773	≥65	To examine the occurrence and associated factors of suspected dementia in elderly patients receiving primary health care.	Urban and rural	BCSI-D	Score ≤4	Dementia 26.8%
*Hu et al. [* 54 *], China*	17 589	≥65	To determine and analyse the population-attributable fractions of changeable risk factors for all types of dementia in rural and urban parts of China.	Urban and rural	MMSE	2018 Chinese guidelines for the diagnosis of dementia	Dementia 9.11%
*Chen et al. [* 55 *], China*	19 276	≥65	To explore how marital status is linked to CI and to compare the impact of marital status on dementia in men and women.	Urban and rural	MMSE, CDR	DSM-IV	Dementia 7.6% for the married group, 16.2% for divorced/separated/widowed
*Yang et al. [* 56 *], China*	925	≥65	To examine how widespread MCI is and to research the understanding of MCI in relation to symptom prevention and intervention.	Suburban	CDR, MoCA-B	DSM-IV	MCI 29.8%, dementia 11.1%.
Southeast Asia							
*Saw et al. [* 57 *], Myanmar*	757	≥60	To to determine the incidence of CI and its risk factors among older adults in Myanmar.	Rural	Myanmar HDS-R	The HDS-R score ≤20 is considered as reduced cognitive function	CI 29.9%, males 23.6%, females 32.9%
*Ganapathy et al. [* 58 *], Malaysia*	3774	≥60	To ascertain the frequency of dementia in Malaysia and to identify factors that affect the quality of life of caregivers for people living with dementia.	Rural	IDEA	Score ≤10	Dementia 8.5%
South Asia							
*Naheed et al. [* 59 *], Bangladesh*	2795	≥60	To estimate how common dementia is among the elderly population in Bangladesh.		MMSE	MMSE<24/30	Dementia 8.0%
*Kumari et al. [* 60 *], India*	440	>60	To discover the frequency and causes of CI and depression in the rural elderly population.	Rural	MMSE	Score 18–23 (mild CI), 0–17 (severe CI)	CI 36%
*Jadenur et al. [* 61 *], India*	150	≥50	To estimate the occurrence of CI in individuals aged ≥50 in the rural area of Belagavi Taluka.	Rural	ACE-III	Score 11–12 (abnormal)	CI 14%
*Khanna et al. [* 62 *], India*	770	≥60	To determine the frequency of CI in the elderly population living in an urban area.	Urban	MMSE	Score <24	CI 8.4%
*Achary et al. [* 63 *], India*	365	≥60	To assess the frequency of MCI among elderly individuals and to investigate the factors linked with MCI.	Urban	HMSE, MoCA-B	MoCA-B score between 19–25 indicative of MCI	MCI 9.3%
*Iype et al. [* 64 *], India*	366	≥65	To determine the occurrence of MCI within a rural setting in Kerala, India.	Rural	Clinical examinations and a semi-structured questionnaire survey	European Consortium on Alzheimer Disease (MCI), DSM-5 (dementia)	MCI 18.6%, dementia 6.8%
*Saldanha et al. [* 65 *], India*	179	x̄ = 75.02	To investigate the prevalence of dementia and its correlation with sociodemographic factors and psychiatric morbidity among residents of old age homes.	Urban	MMSE	ICD-10	Dementia 22.9%
Central Asia							
*Tsoy et al. [* 66 *], Kazakhstan*	668	≥60	To delineate the occurrence and risk factors associated with MCI within an urban population in Kazakhstan.	Urban	MoCA-B	Score ≤26 indicating possible MCI	MCI 30.4%

ACE-III – Addenbrooke’s Cognitive Examination-III, BCSI-D – Brief Community Screening Instrument for Dementia, CDR – clinical dementia rating, CI – cognitive impairment, CMMSE – Chinese Mini-Mental State Examination, DSM-III – Diagnostic and Statistical Manual of Mental Disorders (3rd edition), DSM-IV – Diagnostic and Statistical Manual of Mental Disorders (4th edition), ICD-10 – International Classification of Diseases (10th revision), HDS-R – Revised Hasegawa’s dementia scale, HMSE – Hindi Mental State Examination, IDEA – Identification and Intervention for Dementia in Elderly Africans, MCI – mild cognitive impairment, MMSE – Mini-Mental State Examination, MoCA-B – Montreal Cognitive Assessment (basic version), NIA-AA – National Institute on Ageing and the Alzheimer Association, NINCDS-ADRDA – National Institute of Neurological and Communicative Diseases and Stroke/Alzheimer Disease and Related Disorders Association criteria, SD – standard deviation, SPMSQ – Short Portable Mental Status Questionnaire, x̄ – mean

**Figure 4:**
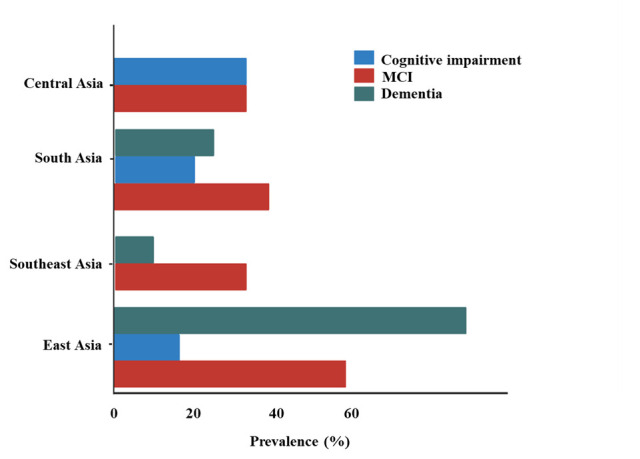
Prevalence of cognitive impairment, mild cognitive impairment, and dementia in Asia by region.

**Figure 4** shows the comparative prevalence rates (%) of cognitive impairment, MCI, and dementia in four major regions of Asia – East Asia, Southeast Asia, South Asia, and Central Asia. Differences across the regions are noticeable as East Asia and Southeast Asia show significantly higher levels of cognitive impairment and dementia compared to South and Central Asia. It is important to note that the lower reported rates of prevalence of dementia in Central Asia may be due to a lack of studies or insufficient data collection in the region rather than a true lower prevalence of dementia. Expanding research efforts in Central Asia is crucial to obtaining accurate data on the prevalence of dementia.

### Prevalence of dementia

Recent studies have revealed a diverse and regionally varied prevalence of dementia across Asia. In Taiwan, a notable study conducted in 2022 with 6549 participants found a high prevalence of 87.1% [29]. This finding was echoed in another Taiwanese study, which identified higher dementia rates in rural areas (8.69%) compared to urban areas (4.46%) [30]. A separate study focusing on institutional residents in Taiwan reported a similar prevalence rate of 87.2% [31]. In contrast, Hong Kong presented a significantly lower prevalence of 41.4% in a 2023 study conducted in urbanised areas [32]. The situation was markedly different in Japan, where a broad age range study (20–97 years) reported a lower prevalence of 6.44% [33], and a four-stage cross-sectional study observed an increasing trend in prevalence of dementia over time, from 4.5% in 1997 to 9.5% in 2016 [34].

**Figure 5:**
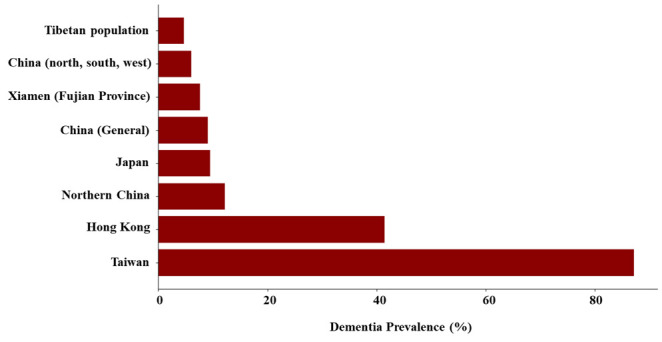
Prevalence of dementia in various countries/regions.

Some countries showed higher average rates, which could indicate demographic differences, such as older average population age or differences in health care systems, diagnostic criteria, and public health policies related to ageing and dementia care. In mainland China, the findings showed considerable variation; for example, a study on the Tibetan population reported a prevalence of 5.12% in males and 4.28% in females [50], while other regions such as Fujian and rural north China showed higher rates of 7.62% [51] and 12.2% [38] respectively. A 2023 Wuhan study highlighted a prevalence of 26.8% among adults aged ≥65 years [53], and in Shanghai’s suburban areas, the prevalence was 11.1% [56]. Furthermore, a comprehensive 2022 study across rural and urban areas in China reported a prevalence of 9.11% [54], and a Hunan Province study in 2023 showed a prevalence of 7.6% [55] (**Figure 5**).

In India, a 2021 study in urban old age homes indicated a 22.9% prevalence [65], contrasting with a 6.8% prevalence found in a 2023 rural Kerala study [64]. Moreover, a 2020 Malaysian study reported an 8.5% prevalence [58], and a 2023 Bangladeshi study recorded an 8.0% prevalence among individuals aged ≥60 years [58].

These findings collectively underline the significant yet varied impact of dementia across Asia, emphasising the influence of geographic, demographic, and methodological factors on the understanding and management of this condition in different settings.

### Cognitive impairment prevalence

**Figure 6:**
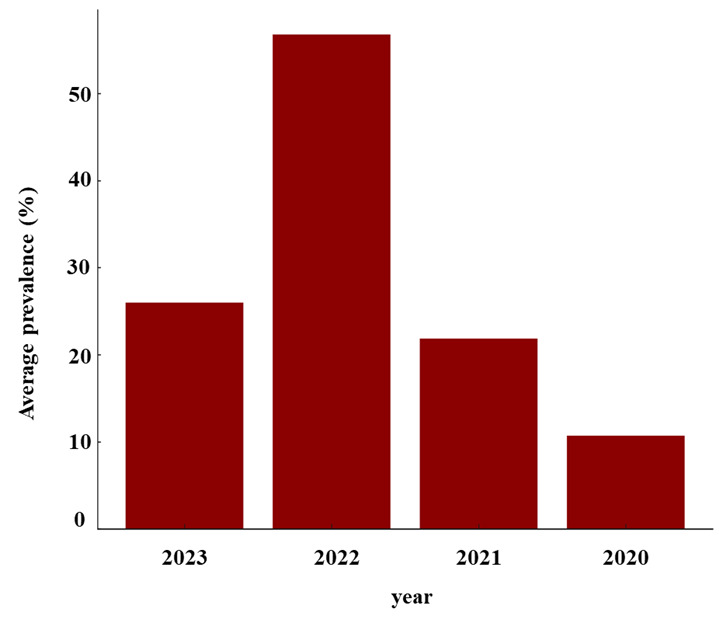
The average prevalence of cognitive impairment by year.

The bar chart in **Figure 6** shows the average prevalence of cognitive impairment across different years. The data spans several years, illustrating fluctuations in the prevalence rates of cognitive impairment during this period. The prevalence of cognitive impairment varies by year, indicating that factors influencing cognitive health may change over time or that different population samples and methodologies in each study year might impact the reported prevalence rates. These fluctuations underscore the complexity of factors influencing cognitive health, including demographic changes, health care access, and societal impacts on mental well-being.

The prevalence and determinants of cognitive impairment among the elderly in Asia, particularly in countries like China, India, and their neighbouring countries, have been a major focus of recent studies. A 2020 study in rural Myanmar reported a significant 29.9% prevalence of cognitive impairment using Hasegawa’s Dementia Scale [57]. In contrast, a 2023 study in Taiwan showed a prevalence of 2.3%, as measured by the Short Portable Mental Status Questionnaire, demonstrating the variability across regions [28]. Gender differences in cognitive impairment were notably observed in North China, with higher rates in females (45.1%) compared to males (40.0%) as assessed by the Mini-Mental State Examination [35]. In India, the prevalence varied. A 2021 study from North India found a 36% prevalence of cognitive impairment and depression in rural elderly [60], whereas a 2022 study in Belagavi Taluka reported a 14% prevalence in those aged >50 years [61]. Urban areas also showed considerable rates, like the 8.4% prevalence found in a 2020 Belagavi District study [62].

According to studies from mainland China, cognitive impairment has been linked to various factors. A 2023 Shanghai study associated it with dietary supplements like folic acid and vitamins, reporting a high 53.8% cognitive impairment rate [36], while a 2020 Qingdao study connected it to dietary habits, reporting a 25% rate in rural elderly [42].

**Figure 7:**
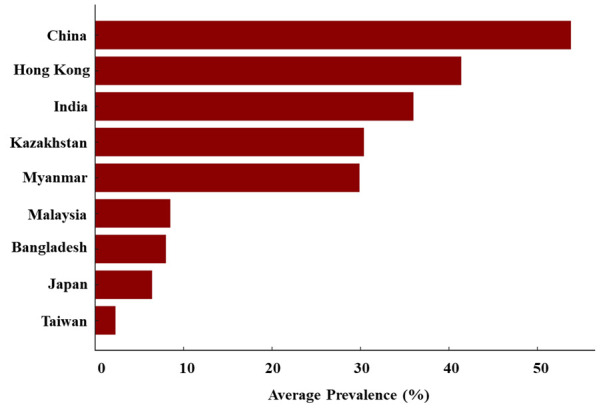
Prevalence of cognitive impairment in various countries/regions.

Other notable findings include a 26.47% prevalence in Shandong Province [37], 24.4% in Jinan [39], and diverse rates in studies assessing larger populations, like 21.48% in a 2023 study of 2598 elderly [46], and 10.7% in a 2020 Tianjin study of 4943 adults [47]. There is a significant variation in cognitive impairment rates across different geographical locations. For instance, China shows a notably high average prevalence rate, contrasting with much lower rates observed in countries/regions like Taiwan and Japan (**Figure 7**).

Urban settings were examined in a 2023 Delhi study, which found a 9.35% prevalence of MCI using the Hindi Mental State Examination and Montreal Cognitive Assessment, basic version [63]. Noteworthy is the high prevalence of MCI among the elderly in a 2021 Beijing study (12.4%) [48], and rural China studies report a prevalence of 11.70% [49] and 23.16% [43]. A 2022 study highlighted an alarming 90.9% cognitive impairment rate in older adults at higher altitudes [41]. Moreover, a 2021 comparative study between urban and rural areas in China reported a 27.8% prevalence of mild cognitive impairment [44], and a 2022 Northern China study linked hypertension and medication adherence to an 18.1% prevalence in the rural elderly [45].

**Figure 8:**
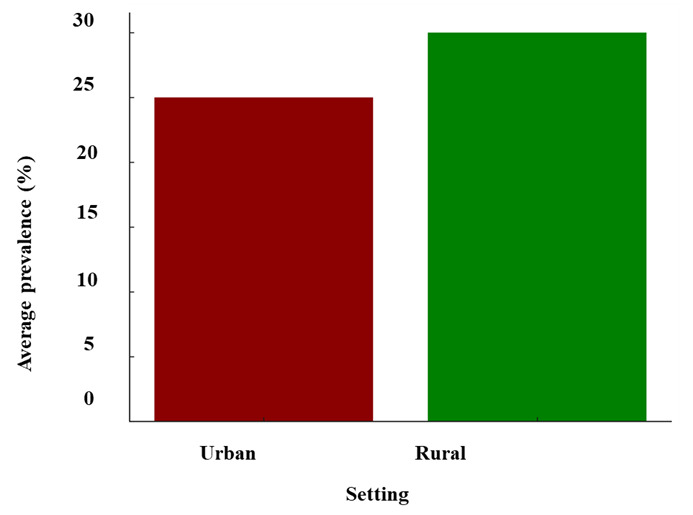
Cognitive impairment prevalence: urban vs rural.

Rural areas had a slightly higher average prevalence rate compared to urban areas (**Figure 8**). This underscores potential disparities in access to health care, education, and lifestyle factors between urban and rural environments that could influence cognitive health. While urban settings may benefit from better health care infrastructure and more opportunities for mental engagement, rural areas face unique challenges, such as limited health care access and fewer cognitive stimulation opportunities.

## DISCUSSION

To our knowledge, this is the first systematic review on the prevalence of cognitive impairment and dementia in Asian elderly populations. The substantial increase in studies from this region has shifted the basis of understanding from reliance on expert opinions informed by limited research to more robust evidence derived from the accumulated data. Through a systematic literature review and adherence to stringent inclusion and exclusion criteria, we successfully compiled and analysed data from 39 cross-sectional studies. These studies collectively reveal significant variations in prevalence across different Asian countries and over time, highlighting the dynamic nature of cognitive health challenges in these populations.

Although there are significant differences in gross domestic product, quality of life, average income, political regimes, health care sector stability, and funding [67], comparing Asian countries is valuable due to rapid demographic transitions and ageing populations across all regions [68]. Understanding dementia and cognitive impairment trends in order to develop effective public health strategies requires an understanding of these trends [69]. Comparing different scenarios helps identify common risk factors and intervention opportunities beyond national borders [70].

Notably, older age [71] and female sex [29,35,40,47,55] appeared as significant risk factors for dementia, consistent with worldwide trends and comparable relationships observed in a variety of international research [72]. The distribution of studies across Asian regions was uneven, with a majority from East Asia, followed by South Asia, Southeast Asia, and a solitary study from Central Asia, while West Asia was notably absent. This uneven distribution reflects the varying levels of research focus and health care infrastructure across the continent [73]. Countries like Japan and China, with more extensive research facilities and funding, reported higher prevalence rates. In contrast, data from less researched areas like Central Asia may not fully capture the regional burden. For example, Japan and South Korea showed higher rates of cognitive impairment compared to countries in South Asia, which might be due to better health care access, diagnostic practices, and public health policies [74].

However, these studies are not without limitations. The cross-sectional nature of the studies makes it challenging to discern changes in function and cognition over time, often relying on third-party reports. Additionally, survivor bias may skew the prevalence data, as those more susceptible to cognitive impairment might not survive into advanced age in Asia.

When compared with Europe, the prevalence of dementia (14.6%) and MCI (35.3%) appears to be higher in some Asian urban areas than in European rural areas [75]. This discrepancy could be attributed to different age structures in the older population between these regions [76]. Asia has a higher percentage of adults aged 66–80 years and fewer aged >81 years, which might lead to a lower overall prevalence of these conditions, primarily associated with old age [77].

The prevalence of mild cognitive impairment in other parts of the world ranges from 13–17% in people aged >65 years, comparable to findings in our review [13,78]. Interestingly, studies on migrant communities in Europe or North America show greater rates of cognitive impairment (8–34%) [79]. These studies often lack clear distinctions in ethnic origins, suggesting a need for more focused research on first-generation Asian migrants.

Assessing cognitive change and mental health in Asia presents unique challenges [80]. In Asian societies, social impairment is not as closely associated with cognitive impairment as in Western countries [81]. This difference may be due to cultural variations in the perception of social roles and expectations for the elderly [82]. In Western contexts, social impairment often closely correlates with cognitive decline because social engagement is a significant aspect of well-being and daily functioning [83]. However, in many Asian cultures, social roles and expectations for the elderly can differ, potentially leading to a less direct association between social impairment and cognitive decline [84]. As a result, screening tools like the Diagnostic and Statistical Manual of Mental Disorders (4th edition) and International Classification of Diseases (10th revision), which incorporate social impairment as a criterion for dementia diagnosis, might lead to underdiagnoses in Asia [85]. The Community Screening Instrument for Dementia, which combines participant-based and informant-based cognitive tests, shows promise as a culturally adaptable tool, although further refinement is needed [86–88].

Large cross-sectional surveys of both rural and urban Asian populations, as well as longitudinal research studying the incidence and moderators of cognitive decline, are required to gain a more comprehensive understanding. Qualitative analyses exploring cultural perceptions of dementia can provide deeper insights into access and health care improvements [89]. Asia faces significant health challenges due to its high mortality rates and the persistent burden of infectious diseases [90]. Additionally, the continent’s rapidly ageing population is expected to lead to an increase in rates of dementia and cognitive impairment [91]. With no known cures or preventative therapies, dementia and cognitive impairment are expected to become serious public health issues throughout Asia in the 21st century [92].

### Recommendations and strategies to adopt

It is essential to implement comprehensive strategies to tackle dementia and cognitive impairment across Asia. This approach requires extensive, long-term studies in both rural and urban populations. Research in this area is crucial for understanding how cognitive decline varies across different regions, as well as identifying the factors that influence these variations. The diversity of cultures across Asia means that the diagnosis and management of dementia must be sensitive to cultural differences. Developing diagnostic tools like Community Screening Instrument for Dementia and Diagnostic and Statistical Manual of Mental Disorders (4th edition) adaptable to various cultural contexts and conducting qualitative analyses to explore how dementia is perceived in different cultures can significantly improve health care access and quality. These analyses are not just beneficial for understanding but are also vital in tailoring health care improvements to specific regional needs.

### Limitations

This research has some limitations. First, all the studies reviewed used a cross-sectional design, which restricts the ability to monitor changes in cognitive function over time. This makes it challenging to comprehend the trajectory of cognitive decline and the lasting effects of different factors on prevalence of dementia.

It is essential to consider potential survivor bias, as people who have lived longer may have different risk factors or protective factors for dementia, which could affect the reported prevalence rates. Additionally, the uneven distribution of studies across different regions is a significant limitation. For example, there is a lack of data from specific areas like West Asia and some parts of Central Asia, which makes it difficult to generalise the findings across the entire continent. Moreover, the variability in diagnostic criteria and study methodologies used across different countries adds to the challenge of comparing prevalence rates and drawing comprehensive conclusions. Future studies should focus on expanding research efforts in underrepresented regions and adopting more consistent methodologies to provide a more accurate and comprehensive understanding of the prevalence of dementia in Asia.

## CONCLUSIONS

In this review, we highlighted the substantial yet varied impact of dementia and cognitive impairment across the Asian continent. We emphasise the necessity of a more holistic understanding of these conditions, considering geographic, demographic, and methodological factors. Consistent with global trends, older age and female sex have been considered key risk factors for dementia. Finally, it is crucial to consider the broader context of these findings. Old age is a significant factor in the increasing prevalence of dementia and cognitive impairment. This situation calls for urgent and tailored public health strategies, particularly given the fact that the prevalence of MCI and dementia in some Asian urban areas is higher than that in European rural areas; it is essential to understand the factors contributing to these differences. This comparison highlights that urban environments in Asia may face unique challenges, such as rapid population growth, high levels of environmental stress, and varying health care access, which could contribute to higher rates of cognitive decline. The development of these strategies must be informed by the nuanced understanding of the regional variations and cultural specificities of dementia and cognitive impairment in Asia.

## Additional material


Online Supplementary Document
