# Supplementary material for: Emerging trends in cognitive impairment and dementia among older populations in Asia: A systematic review
Source: J Glob Health. 2024 Nov 8;14:04233. doi: 10.7189/jogh.14.04233 (PMC11544519; doi:10.7189/jogh.14.04233)
Supplement: Online Supplementary Document [file jogh-14-04233-s001.pdf]

Table S1. Risk of Bias of the Included Studies by JBI-MAStARI

**Author, Year, Ref**	**Q1**	**Q2**	**Q3**	**Q4**	**Q5**	**Q6**	**Q7**	**Q8**	**Yes Score (_/8) Methodological quality**
Lor Y-CM. et al. 2023 [25]	Y	Y	Y	Y	Y	Y	N	Y	7/8
Kao YH. et al. 2022 [26]	Y	Y	Y	Y	Y	Y	Y	Y	8/8
Liu CC. et al. 2022 [27]	Y	Y	Y	Y	Y	Y	N	Y	7/8
Hsieh SW. et al. 2021 [28]	Y	Y	Y	Y	Y	Y	N	Y	7/8
Ho HC. et al. 2023 [29]	Y	Y	Y	Y	Y	Y	Y	Y	8/8
Takenoshita S. et al. 2023 [30]	Y	Y	Y	Y	Y	Y	N	Y	7/8
Shimizu H. et al. 2022 [31]	Y	Y	Y	Y	Y	Y	Y	Y	8/8
Wang J. et al. 2020 [32]	Y	Y	Y	Y	Y	Y	N	Y	7/8
Jiang X. et al. 2023 [33]	Y	Y	Y	Y	Y	Y	Y	Y	8/8
Cong L. et al. 2022 [34]	Y	Y	Y	Y	Y	Y	Y	Y	8/8
Gan J. et al. 2022 [35]	Y	Y	Y	Y	Y	Y	Y	Y	8/8
Jiang F. et al. 2021 [36]	Y	Y	Y	Y	Y	Y	Y	Y	8/8
Liu Y. et al. 2022 [37]	Y	Y	Y	Y	Y	Y	Y	Y	8/8
Liu S. et al. 2022 [38]	Y	Y	Y	Y	Y	Y	Y	Y	8/8
Xu R. et al. 2020 [39]	Y	Y	Y	Y	Y	Y	Y	Y	8/8
Xue J. et al. 2022 [40]	Y	Y	Y	Y	Y	Y	Y	Y	8/8
Liu D. et al. 2021 [41]	Y	Y	Y	Y	Y	Y	Y	Y	8/8
Ma LY. et al. 2022 [42]	Y	Y	Y	Y	Y	Y	Y	Y	8/8
Xu T. et al. 2023 [43]	Y	Y	Y	Y	Y	Y	Y	Y	8/8
Fu J. et al. 2020 [44]	Y	Y	Y	Y	Y	Y	Y	Y	8/8
Fan R. et al. 2021 [45]	Y	Y	Y	Y	Y	Y	Y	Y	8/8
Duan H. et al. 2021 [46]	Y	Y	Y	Y	Y	Y	Y	Y	8/8
Mima D. et al. 2023 [47]	Y	Y	Y	Y	Y	Y	Y	Y	8/8
Song Y. et al. 2023 [48]	Y	Y	Y	Y	Y	Y	Y	Y	8/8
Jia L. et al. 2020 [49]	Y	Y	Y	Y	Y	Y	Y	Y	8/8
Wang ZQ. et al. 2023 [50]	Y	Y	Y	Y	Y	Y	Y	Y	8/8
Hu FF. et al. 2022 [51]	Y	Y	Y	Y	Y	Y	Y	Y	8/8
Chen ZC. et al. 2023 [52]	Y	Y	Y	Y	Y	Y	Y	Y	8/8
Yang J. et al. 2021 [53]	Y	Y	Y	Y	Y	Y	Y	Y	8/8
Saw YM. et al. 2020 [54]	Y	Y	Y	Y	Y	Y	Y	Y	8/8
Ganapathy SS. et al. 2020 [55]	Y	Y	Y	Y	Y	Y	Y	Y	8/8
Naheed A. et al. 2023 [56]	Y	Y	Y	Y	Y	Y	Y	Y	8/8

Author, Year, Ref	Q1	Q2	Q3	Q4	Q5	Q6	Q7	Q8	Yes Score (_/8) Methodological quality
Kumari R. et al. 2021 [57]	Y	Y	Y	Y	Y	Y	Y	Y	8/8
Jadenur SS. et al. 2022 [58]	Y	Y	Y	Y	Y	Y	Y	Y	8/8
Khanna AB. et al. 2020 [59]	Y	Y	Y	Y	Y	Y	Y	Y	8/8
Achary MT. et al. 2023 [60]	Y	Y	Y	Y	Y	Y	Y	Y	8/8
Iype T. et al. 2023 [61]	Y	Y	Y	Y	Y	Y	Y	Y	8/8
Saldanha D. et al. 2021 [62]	Y	Y	Y	Y	Y	Y	Y	Y	8/8
Tsoy RT. et al. 2019 [63]	Y	Y	Y	Y	Y	Y	Y	Y	8/8

The items were collapsed into 8 quality-appraisal criteria (Q1-Were the criteria for inclusion in the sample clearly defined? Q2-Were the study subjects and the setting described in detail? Q3-Was the exposure measured in a valid and reliable way? Q4-Were objective standard criteria used for measurement of the condition? Q5Were confounding factors identified? Q6-Were strategies to deal with confounding factors stated? Q7-Were the outcomes measured in a valid and reliable way? Q8Was appropriate statistical analysis used?).

JBI-MAStARI was used to evaluate the risk of bias. Articles with scores between 1 and 2 were defined as poor methodological quality, articles with scores between 3 and 4 as moderate quality, and articles with scores more than 5 as high quality.
N, no; NA = not relevant; U = unsure; Y = yes.
